# The role of calcium channel blockers in wound healing

**DOI:** 10.22038/ijbms.2018.29753.7182

**Published:** 2018-12

**Authors:** Hoda Mojiri-Forushani

**Affiliations:** 1 Department of Pharmacology, Abadan School of Medical Sciences, Abadan, Iran

**Keywords:** Amlodipine, Azelnidipine, Calcium channel blockers, Diltiazem, Nifedipine, Verapamil, Wound healing

## Abstract

Wound healing is a natural response to restore the injured tissue to normal. Wound healing is also complicated process involving different cellular, molecular and biochemical mechanisms and various types of cytokines and growth factors. Calcium channel blockers belong to cardiovascular medicine and administrated to treatment of hypertension, angina and cardiac arrhythmia because of vasodilatory effect. Calcium channel blockers is divided to dihydropyridine and non-dihydropyrine. The potential of both dihydropyridine and non-dihydropyrine calcium channel blockers in wound healing have been reported in different animal models and *in vitro* previously. Amlodipine, verapamil, diltiazem, nifedipine, and azelnidipine are calcium channel blockers that indicated wound healing property. The various mechanisms that involve in wound healing effect of calcium channel blockers are discussed in this article.

## Introduction

Skin wound healing is characterized by three phases, inflammation, proliferation, and remodeling. Wound healing is a complex physiological event that involves different cell types. During the inflammation phase, inflammatory cells, such as cytokines, and growth factors accumulate at the site of injury. Then, fibroblasts secrete collagen and fibronectin as part of the production of the new extracellular matrix. Thereafter, epithelial cells migrate across the wound bed and myofibroblasts contract the wound margins. Finally, the remodeling phase leads to maturation of the granulation tissue into mature connective tissue or scar. Any change to the wound repair process may result in incomplete or delayed healing (1). 

Calcium channel blockers (CCBs) are considered key agents for the treatment of cardiovascular diseases, such as hypertension, angina pectoris, and cardiac arrhythmias (2). Some studies have shown CCBs to have beneficial effects in other conditions, such as wound healing. In animal model studies, verapamil, diltiazem, nifedipine, and azelnidipine have been suggested to play potential roles in wound healing (3-5). In a previous study, a dihydropyridine CCB, amlodipine, was shown to ameliorate wound healing and decrease the healing time (6). 

Evidence has shown that CCBs have antioxidant properties and increase collagen accumulation and fibroblast proliferation through the promotion of nitric oxide (NO) production (7). NO plays an essential role in angiogenesis and in the proliferation of fibroblasts, epithelial cells, and keratinocytes, during wound healing. Tissue collagenases, such as matrix metalloproteinases, are expressed more abundantly at tissue injury sites; their action depends on the associated inflammatory cytokines and intracellular calcium levels (8). 

Furthermore, CCBs are known to have vasodilatory properties, increasing blood flow to the wound area and stimulating growth factor production (9). Conversely, gingival hyperplasia is a side effect of CCB use. Both inflammatory and non-inflammatory pathways may lead to gingival hyperplasia. In the inflammatory pathway, the probable up-regulation of some cytokines (e.g., transforming growth factor-β) also benefits healing. In the non-inflammatory mechanism, collagenase activity is decreased due to reduced folic acid uptake (10).

## Conclusion

CCBs typically demonstrate relatively minor side effects and are associated with good patient compliance in a variety of clinical conditions. Additionally, they may, in the future, be introduced as new and promising wound healing treatments.

## Conflicts of Interest

The author declare that no conflict of interest exist.
